# Extracellular Vesicles As Modulators of Tumor Microenvironment and Disease Progression in Glioma

**DOI:** 10.3389/fonc.2017.00144

**Published:** 2017-07-05

**Authors:** Abir Mondal, Divya Kumari Singh, Suchismita Panda, Anjali Shiras

**Affiliations:** ^1^Lab-3, RNA Biology and Cancer Laboratory, National Centre for Cell Science, S.P. Pune University Campus, Pune, India

**Keywords:** glioblastoma, microenvironment, angiogenesis, microRNAs, extracellular vesicles

## Abstract

Diffuse gliomas are lethal tumors of the central nervous system (CNS) characterized by infiltrative growth, aggressive nature, and therapeutic resistance. The recent 2016 WHO classification for CNS tumors categorizes diffuse glioma into two major types that include IDH wild-type glioblastoma, which is the predominant type and IDH-mutant glioblastoma, which is less common and displays better prognosis. Recent studies suggest presence of a distinct cell population with stem cell features termed as glioma stem cells (GSCs) to be causal in driving tumor growth in glioblastoma. The presence of a stem and progenitor population possibly makes glioblastoma highly heterogeneous. Significantly, tumor growth is driven by interaction of cells residing within the tumor with the surrounding milieu termed as the tumor microenvironment. It comprises of various cell types such as endothelial cells, secreted factors, and the surrounding extracellular matrix, which altogether help perpetuate the proliferation of GSCs. One of the important mediators critical to the cross talk is extracellular vesicles (EVs). These nano-sized vesicles play important roles in intercellular communication by transporting bioactive molecules into the surrounding milieu, thereby altering cellular functions and/or reprogramming recipient cells. With the growing information on the contribution of EVs in modulation of the tumor microenvironment, it is important to determine their role in both supporting as well as promoting tumor growth in glioma. In this review, we provide a comprehensive overview of the role of EVs in tumor progression and glioma pathogenesis.

## Introduction

Adult diffuse gliomas are histopathologically categorized into grades II–IV as oligodendroglioma, oligo-astrocytoma, astrocytoma, and glioblastoma (1). Glioblastoma are highly aggressive and angiogenic tumors with median survival of 12–15 months. To ensure appropriate treatment, it is important to correctly grade glial tumors. However, due to the inter- and intraobserver variability encountered in histopathological analyses of gliomas, tumor grading based on gene expression and DNA methylation signatures is gathering importance. The recent glioma classification is based on the status of mutations of isocitrate dehydrogenase genes 1 and 2 (IDH1/2), 1p/19q co-deletion, ATRX alterations, and TERT promoter mutation status (2). Using TCGA data that consider genomic signatures, survival time, patient age, and treatment responses, glioblastoma is further subclassified into proneural (PN), neural (N), classical (C), and mesenchymal (MES) subtypes. Here, genomic aberrations in expression of EGFR, NF1, and PDGFRA/IDH1 define the classical, MES, and PN subtypes, respectively (3). MES subtypes demonstrate poor survival as compared to PN, thereby necessitating determination of correct molecular signatures in glioblastoma (3).

A predominant feature of glioblastoma is the high level of inter- and intracellular heterogeneity, due to the presence of cell population within the tumor that shows various stages of differentiation. Glioblastoma is considered to be propagated by a specific subpopulation of glioma stem cells (GSCs) that are responsible for therapeutic resistance and recurrence (4–7). The GSCs maintain two mutually exclusive molecular identities, i.e., PN or MES. Following therapy, GSCs display phenotypic transition from Proneural to MES subtype leading to tumor progression and increased aggressiveness. Also, the tumor microenvironment contributes toward a MES signature in glioblastoma (8). Interestingly, the stroma in which the GSC pool resides is considered to be the GSC niche and is responsible for tumor aggressiveness. The niche can either be a perivascular niche in which GSCs reside in close proximity to the tumor vasculature or a niche invaded by GSCs where cancer cells co-opt normal blood vessels enabling their migration into brain parenchyma or a hypoxic tumor niche (9). Angiogenesis is induced by the production of high levels of proteins such as VEGF, FGF, and PDGF by glioma cells (10). These factors induce proliferation of endothelial cells that not only help to recruit bone marrow-derived endothelial cells and pericyte precursors but also cause cancer cells to transdifferentiate into endothelial cells or pericytes (11, 12). This may disrupt the blood–brain barrier (BBB) and lead to treatment failure. The GSCs support growth and the infiltrative character of other cancer cells in a paracrine and autocrine manner by secreting angiocrine factors, cytokines, and chemokines (13). In a hypoxic microenvironment, this creates a permissive atmosphere for malignant progression (14, 15). Several mechanisms exist that help mediate cross talk of GSCs and the surrounding tumor microenvironment. Prominent among them is communication of cancer cells with the outside environment (within its microenvironment and even at distant sites) through extracellular vesicle (EV)-mediated transport. In summary, tumor propagation is a cumulative effect of the GSC population and their communication with the microenvironment that includes the tumor vasculature, immune cells, and non-stem cells. This complex biological network arising from intracellular, intercellular, and distant cell interactions supports growth of aggressive and therapy-resistant glioblastoma tumors.

Molecules that are important in reprogramming, metabolism, and angiogenesis are packaged into EVs and transported to proximal or distant cells, affecting proliferation and angiogenesis (16). These EVs serve as carriers of various types of molecules such as lipids, proteins, mRNAs, miRNAs, long non-coding RNAs (lncRNAs), and DNA. EVs can also directly activate cell surface receptors *via* bioactive ligands and transfer these to neighboring cells, along with transcription factors, oncogenes or infectious particles (17), and modulate tumor microenvironment (Table 1). In this review, we elaborate on the role of EVs in glioblastoma pathogenesis.

**Table 1:** Composition of putative biomolecules in glioblastoma-derived EVs and their respective functions.

Physiological and pathophysiological functions	Biomolecules exported by extracellular vesicles		Reference
Tumor growth, metabolism, invasion, and metastasis	Proteins	EGFRvIII	(18)
		Trk β	(19)
		MMPs, PDGFs, caveolin 1, lysyl oxidase, IL-8	(20)
		Annexin A2	(21)
		CLIC1	(22)
		Semaphorin 3A	(23)
	mRNAs	EGFR	(18)
		Podoplanin	(24)
		Mutant IDH1	(25)
	ncRNAs	miR-15b, 16, 19b, 21, 26a, 92	(16)
		miR-1	(21)
		miR-27b, 451, 222, 135b, 30e, 451	(26)
Immune suppression	Proteins	TGF-β	(27)
		IL-6	(16)
Angiogenesis	Proteins	Angiogenin, IL-VEGF, and tissue factor	(16, 28)
	ncRNAs	miR-19b	(16)
		Linc-POU3F3	(29)
Therapy resistance	Proteins	Trk β	(19)
		IL-6	(16)
	mRNAs	MGMT, APNG, EGFR, CD63, ERCC1	(30)
		TIMP1, TIMP2	(31)
	ncRNAs	miR-21	(16, 32)
		miR-100	(26)
		miR-221	(33)
Biomolecules with unknown functions	ncRNAs	miR-27a, 92, 93, 320, 20	(16)
		RNU6, miR-483-5p, 574-3p, 197, 484, 146a, 223	(34)
		miR-451a, 4301, 5096, 3676-5p, 4454, 1303, 1273a, 619, 448, 1246, 4792, 5095, 1273g, 4256, 4255, 5100, 1285-1, 1269b, 4500, 1273d, 4443 let-7b, 9a, 30a, 30d, 30b, 22, 125a, 25, 29a, 4301, 27b, 23b, 5096, 3676, 374b, 339, 191, 4454	(26)
		miR-24, 103, 125	(35)
	DNA	Mitochondrial DNA	(36)
Diagnostic marker	gDNA	IDH1^G395A^gDNA	(37)

*mRNA, messenger RNA; ncRNA, non-coding RNA; gDNA, genomic DNA; miR, microRNA*.

## EV Structure, Biogenesis, and Molecular Contents

The EVs are phospholipid bilayer-enclosed vesicles secreted by various cell types displaying a size range between 30 and 1,000 nm. They are broadly categorized into microvesicles (MVs, up to 1,000 nm in diameter) and exosomes (30–100 nm) based on their size, intracellular origin, and biogenesis pathway (38, 39). Characteristically, the MVs are formed by outward budding and fission of the cell membrane, whereas exosomes are of endosomal origin (38). The multivesicular body (MVB) formation occurs either through the endosomal sorting complex required for transport (ESCRT) machinery or *via* an ESCRT-independent manner. The ESCRT machinery consists of four complexes of approximately 30 proteins that are responsible for sequestering ubiquitinated transmembrane proteins in the endosomal membrane followed by their excision in the form of sorted cargo by budding (40). The ESCRT-independent manner is mediated *via* tetraspanin CD63 and enzymes sphingomyelinase, and phospholipase D2 (41, 42). Baietti et al. showed that the heparin sulfate proteoglycan syndecan and its cytoplasmic adaptor syntenin have roles in exosome formation (43). Several posttranslational modifications are involved in the sorting of specific proteins into exosomes, like SUMOylation of heterogeneous nuclear ribonucleoproteins A2/B1 that promotes the sorting of specific microRNAs into exosomes and also regulates sorting of α-synuclein into EVs (44, 45).

Interestingly, exosome secretion is mediated through SNARE and Rab proteins (RAB7, RAB11, RAB27, and RAB35) (46). The release of EVs followed by their uptake in recipient cells and delivery of cargo may occur in various ways. It occurs either by direct fusion of EVs with the plasma membrane of recipient cells or through fusion with the endosomal membrane following acidification (47). Hsu et al. demonstrated that Rab3 helps in exosome secretion by facilitating the docking and tethering of MVBs to the plasma membrane (48). Non-canonical Wnt5a-Ca++ signaling was shown to induce release of exosomes into the extracellular environment of melanoma cells (49). Interestingly, the release of exosomes by tumor suppressor activated pathway 6 (TSAP6) gene occurs in a p53-dependent manner (50). Another posttranslational modification, ISGylation was shown to be important in the control of exosome production ISGylation of MVB proteins such as TSG101 regulated exosome release by triggering MVB colocalization with lysosomes and promoted degradation of MVB proteins (51). Although the formation of MVs is controlled by ADP-ribosylation factor 6 and membrane lipid microdomains (52), mechanisms responsible for sorting of cargo into the lumen of MVBs that form exosomes are not fully understood (53).

## Role of EVs in Cellular Cross Talk and Glioblastoma Progression

**Figure 1:**
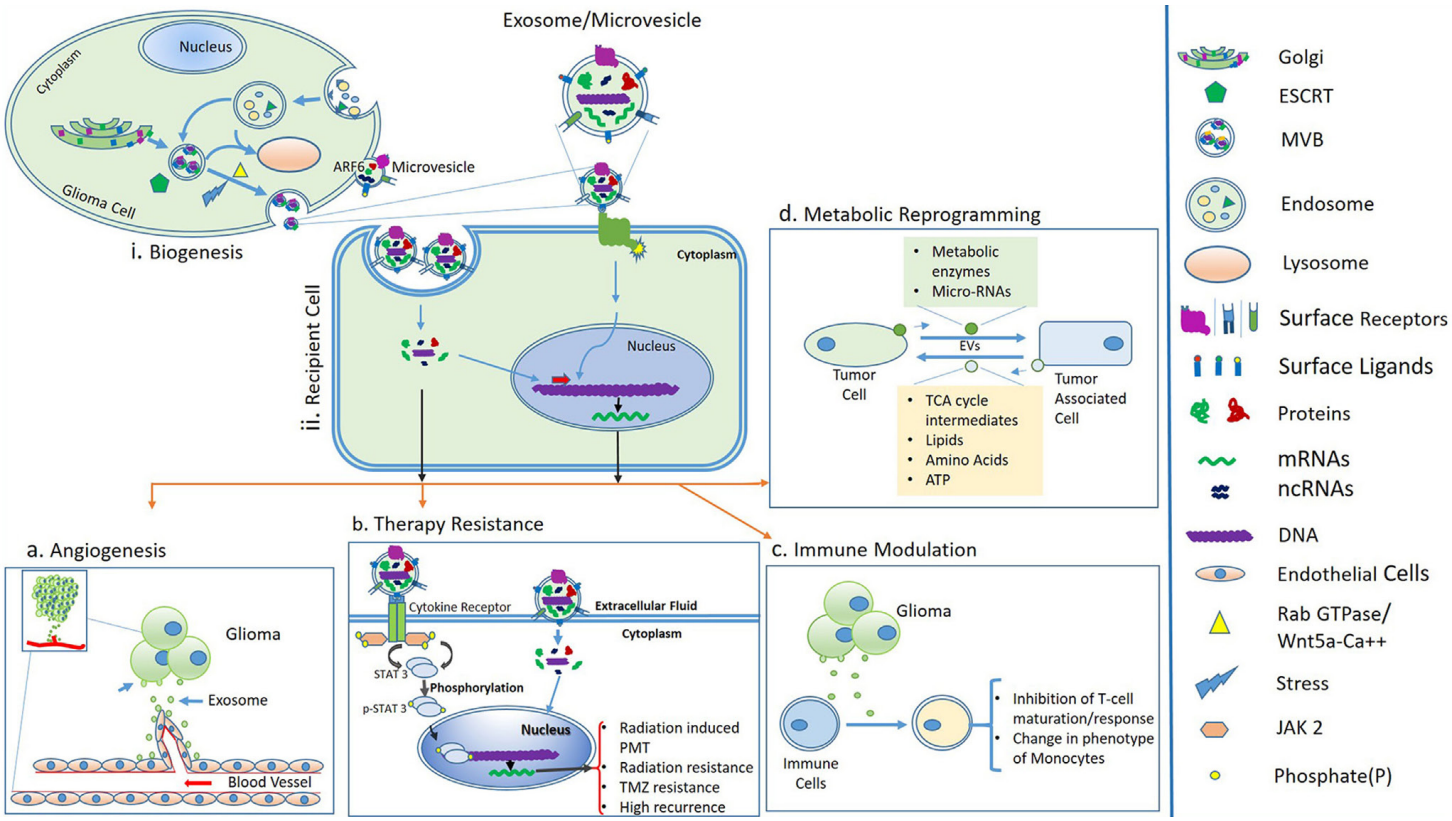
(i) Biogenesis and secretion of extracellular vesicles (EVs) such as MVs and exosomes. Sorting of cargo molecules in multivesicular bodies (MVBs) occur in an endosomal sorting complex required for transport (ESCRT)-dependent manner. Exosomes are of endosomal origin and their secretion is mediated by Rab GTPase family proteins and the Wnt5a-Ca++ non-canonical pathway. In an alternative pathway, the release of MVs is governed by ADP-ribosylation factor 6 (ARF6) and membrane lipid microdomains. (ii) Uptake of EVs by recipient cells or binding of surface ligands of EVs to recipient cells is followed by downstream molecular cascades resulting in processes like angiogenesis, therapy resistance, immune modulation, and metabolic reprogramming, **(A)** angiogenesis; tumor-derived EVs modulate the formation of blood vessels, which supports glioma progression, **(B)** therapy resistance; exosomes or MVs carry cytokines, which may further activate STAT3 protein *via* cytokine receptors and ultimately leads to proneural–mesenchymal transition (PMT) and a radiation-resistant phenotype of glioma (56). Activation of STAT3 signaling also promotes temozolomide resistance of glioma (57). **(C)** Immune modulation; glioma-derived exosomes are able to inactivate immune responses by inhibiting T-cell maturation and changes in phenotypes of monocytes and **(D)** metabolic reprogramming; possible through transfer of metabolic enzymes to tumor-associated cells *via* EVs and in turn tumor cells acquire energy and nutrients, which support glioma growth.

Tumor-derived EVs act as a multicomponent delivery vehicle to transfer genetic information as well as signaling proteins to cells in their vicinity as well as at distant sites (Figure 1). Numerous functions are attributed to EVs in cancer that range from their role in antitumor immunity, drug resistance, metastasis, angiogenesis, and intercellular communication to reprogramming (54). Reprogramming is a process of conversion of differentiated cells into a dedifferentiated state and can be mediated by MVs in *in vivo* conditions (55).

Glioblastoma-derived MVs are likely to represent one of the mechanisms by which cancer cells change the tumor microenvironment and make it more permissive for growth and invasion (58). Therefore, it is worth investigating the molecular cargo present in EVs for early glioma detection. The four glioblastoma subtypes activate different pathways of vesicle formation, and each subtype shows significant differences in expression of the EV regulatory and biogenesis markers (59). The molecules present in EVs of which expression was subtype- specific include CD63, CD81, RAB27A, RAB27B, FLOT1, FLOT2, TSG101, RAB 5A among others (53). Recently, Kowal et al. proposed subcategorization of EVs based on relative abundance of specific EV protein markers such as CD63, CD9, and CD81 (60). Godlewski et al. showed that different subtypes of GSCs show highly heterogenous profiles of miRNAs. Moreover, EV-mediated transfer and secretion of miRNAs may contribute to glioblastoma heterogeneity (61). Importantly, the effect of phenotypic transition of GSCs from PN to MES signature is reflected significantly in the release and content of EVs (62, 63).

The significant contribution of EVs in key cellular processes related to disease progression in glioma is outlined below.

## Metabolic Regulation

Glial tumors show propensity for non-oxidative metabolism of glucose even in the presence of oxygen, a phenomenon known as the Warburg effect (61, 64). Glioblastoma cells were also found to be highly oxidative indicating that substrate oxidation also occurs along with aerobic glycolysis and lactate release (65). The GSCs have other metabolic strategies or substrates as compared to bulk tumor cells. The tumor microenvironment and genetic factors contribute immensely toward metabolic reprogramming in glioblastoma. The hypoxic microenvironment within the tumor results in a shift toward glycolysis and shows angiogenic phenotype whereas tumor cells at the edge are highly invasive and heavily dependent on mitochondrial respiration for energy production (66, 67). Kucharzewska et al. showed that hypoxia-dependent intercellular signaling in glioblastoma is mediated through exosomes (20). They showed that hypoxia was associated with secretion of exosomes enriched in hypoxia-regulated mRNAs and proteins such as matrix metalloproteinases, IL-8, PDGFs, caveolin 1, and lysyl oxidases which performed pivotal roles in cellular metabolism and cell proliferation. In addition, mutations in metabolic genes such as IDH1/2 were important in gliomagenesis and had prognostic importance (68). Khurshed et al. showed that energy metabolism differed between IDH1 wild-type and mutant glioma (69). IDH1 mutant glioma cells used oxidative TCA cycle for metabolism whereas IDH wild-type glioma was more dependent on glycolytic and lactate metabolism. Recently, EVs isolated specifically from cerebrospinal fluid (CSF) contained information regarding the mutational profile of IDH1 in brain tumors (70). Interestingly, several metabolic enzymes were overexpressed in brain tumors, suggesting that the cancer cells derived energy and nutrients needed for proliferation by transferring these enzymes to surrounding cells through EVs under hypoxic conditions. In addition, mitochondrial DNA was also detected in MVs of glioblastoma cells but its function is not yet understood (36).

## Immune Modulation

Tumor-derived MVs were found to be enriched in CD39 and CD73 in various types of cancers such as pancreatic, bladder, and breast cancers. CD39 and CD73 were also highly expressed in gliomas causing adenosinergic immunosuppression (71) but its status in glioma EVs is not known. Glioma-derived MVs were shown to activate myeloid-derived suppressor cells (MDSCs) (72). MDSCs modulate immune activity by inhibiting T-cell responses (73). Moreover, glioblastoma-derived MVs were shown to contain IL-6 that has a role in phosphorylation of STAT-3 on MDSCs, causing immunosuppression. In addition, TGF-β in MVs caused similar effect in gliomas (27). van der Vos et al. showed that glioma-derived EVs transferred miR-451 and miR-21 to microglia/macrophages leading to downregulation of their targets (74), whereas uptake of GSC exosomes by monocytes caused failure to mount an immune response against glioma cells (75, 76). Furthermore, glioma cell-derived exosomes suppressed T-cell immune responses by acting on monocyte maturation rather than affecting their direct interaction with T cells (77). Moreover, glioma-derived MVs were restricted in their capacity to directly prime peripheral immunosuppression (78). Hence, the role of MVs in immune suppression needs further investigation.

## Angiogenesis

Proteins that are expressed under hypoxic conditions, such as HIF are responsible for angiogenesis in glioblastoma (79). Glioblastoma-derived MVs contain VEGF, angiogenin, IL-8, PDGF, and miRNA-19b, and it has been shown that VEGF and angiogenin bind to the cognate receptor on the surface of ECs and promote angiogenesis (16, 20). Instead, miR-19b-mediated angiogenesis by repressing anti-angiogenic proteins such as thrombospondin-1 and connective tissue growth factor within tumors (80). Recently, semaphorin3A was found in the exosomes derived from blood or CSF which acted as a pro-permeability factor but with anti-angiogenic function (23). Interestingly, angiogenesis was also induced in glioma by exosomes enriched in lncRNA, POU3F3 (29). Svensson and Belting demonstrated a significantly increased content of tissue factor (TF) in glioblastoma cell-derived EVs under hypoxic conditions (81). In addition, EVs were also shown to transfer the oncogenic form of EGFR, EGFRvIII, between glioblastoma cells as well as to ECs, causing phenotypic modulation of recipient cells (18). Moreover, EGFRvIII-transformed cells became hypersensitive to TF/protease activated receptor (TF/PAR-2) signaling. This kind of receptor transfer may cause angiogenic signaling in recipient cells due to regulation of VEGF gene expression by EGFRvIII. This suggests that under hypoxic conditions, even in the absence of external stimuli, tumor angiogenesis is supported through PAR-2 in ECs.

## EVs in Tumor Growth, Invasion, and Therapy Resistance

There are several tumor cell resistance mechanisms that affect therapy response in glioblastoma such as

- The cross talk of GSCs with the tumor microenvironment.
- Decreased drug uptake, increased drug efflux and intracellular drug inactivation.
- Repair of drug-induced damage or defects in DNA damage response pathway.

Earlier studies showed that chemoresistance of the CD133+ GSC population was due to upregulation of miR-9-2 and MDR1. The protein target of miR-9-2 was patch homolog1. It is expressed at low levels in temozolomide (TMZ)-resistant CD133+ cells in which Gli1 expression was higher (82). Also, miR-9 was shown to be high in TMZ-resistant cells, and MVs were strongly involved in functional delivery of anti-miR-9 from mesenchymal stem cells to glioblastoma cells, imparting TMZ sensitivity (83). Most drugs such as TMZ and cisplatin are alkylating agents and cause DNA damage by exerting their cytotoxic or mutagenic effects on cells. Epigenetic silencing of the DNA repair gene MGMT by promoter methylation compromised DNA repair and was associated with longer survival in glioblastoma patients treated with alkylating agents (84). In addition, other DNA repair genes such as ERCC1, ERCC2, MUTYH, and PNKP reduced efficacy of alkylating agents, imparting chemoresistance in glioma (85). Shao et al. showed that ERCC1 mRNA levels were upregulated in MVs derived from glioblastoma cells. Also, the mRNA levels of microvesicular MGMT, APNG, or both were elevated in resistant glioblastoma cell lines as compared to sensitive cell lines (30). Hence, increased patient-derived microvesicular MGMT and APNG mRNA levels are indicative of drug resistance or they predict alkylating drug responses in glioblastoma patients. While MGMT promoter methylation is associated with a better prognosis, mutation/amplification of EGFR is associated with poor prognosis (84, 86). EGFRvIII protein is transferred in glioblastoma cell-derived MVs, signifying its role as a prognostic biomarker (18).

Recent studies also highlight the ability of EVs in promoting glioma growth and invasion. TrkB, a member of the neurotrophin tyrosine kinase receptor-1 family was shown to be highly expressed in exosomes of glioblastoma patients and its level correlated with tumor progression and aggressiveness (19). Also, it was shown that differential neurotrophin receptor expression levels displayed by exosomes depended on the differentiation status of tumors and YKL-40 expression, thereby making exosomal TrkB a novel biomarker for glioblastoma. In addition, Timp1 as one of the NF-κB target genes with a role in tumor growth was significantly upregulated in exosomes (31). Recently, a circulating protein, CLIC1 with growth stimulatory properties both *in vitro* and *in vivo* was identified in EVs of GSCs (22). Apart from these molecules enclosed in MVs, a tumor suppressor protein such as PTEN is also exported through exosomes to recipient cells where it suppresses cell proliferation by reducing the abundance of pAkt (87). Inhibition of pAkt levels diminished tumor growth and invasion.

## EVs in Glioma Immune Therapy

Exosomes also serve as an attractive candidate for immune therapy of brain tumors as they retain their stability during purification as well as under *in vivo* conditions. Vaccination with dendritic cell-derived exosomes showed good recovery against malignancy with little adverse effects in phase I and II clinical trials (88, 89). Muller et al. found a negative correlation between mRNA expression levels of TIMP1, TGF-β, and IL-8 in exosomes and patient’s survival after a vaccination trial in glioblastoma patients. Instead, exosomal mRNA levels of cytokines IL-8 and TGF-β, important in glioma growth and metastasis, showed a clear decrease after vaccination (31).

## miRNAs in Glioblastoma EVs

A large number of microRNAs are found to be encapsulated in EVs in serum of glioma patients. While their functions in relation to microenvironment in glioma are still being explored, they certainly pose great hope as circulating biomarkers for early diagnosis, tumor staging and prognostication. miR-21 and miR-221 were shown to be highly enriched in CSF-derived EVs and serum-derived exosomes of glioblastoma patients, respectively, and hence possessed the potential to serve as a relevant biomarker (32, 33). More importantly, the level of miR-221 increased with glioma grades in exosomes. In addition, levels of other miRNAs like miR-24, miR-103, and miR-125 along with miR-21 were also high in exosomes derived from CSF of glioblastoma patients (35). Although several other miRNAs were detected in glioma MVs (Table 1), their mechanism of action in target cell is largely unknown which limits their use in glioblastoma therapy.

## Future Prospects

Exosomes play a critical role in mediating intercellular communication. Being nano-sized and lipid bilayered, they can easily cross the BBB under both physiological as well as abnormal conditions. Moreover, the enclosed biomolecules are stably retained in an active state and are functional after uptake by recipient cells. These characteristics make MVs and/or exosomes candidates for therapeutic applications. Exosomes derived from different cell types can be used to selectively deliver therapeutic nucleic acid analogs (tumor suppressor miRNAs/ncRNA mimics or oncogenic miRNA inhibitors) or conventional drugs for applications in tumor therapy (90). Moreover, the study of molecular cargo of EVs is helpful for the identification of novel biomarkers in disease diagnosis and monitoring (91). The tumor-derived MVs of glioblastoma show upregulation of several signature molecules, which offer rapid discrimination between tumor-derived EVs and normal cell-derived EVs. This simplifies the diagnosis and circumvents the use of invasive methods such as biopsies. Interestingly, the EVs derived from CSFs contain RNA signatures reflective of the underlying molecular genetic status of glioblastoma in terms of wt EGFR expression and EGFRvIII status.

The EVs being more enriched in CSF than in serum are easier to detect using non-invasive tools such as PCR or droplet digital PCR (25). With advances in such technologies, it is possible to identify as well as sub-classify glioblastoma tumors from inaccessible locations. Moreover, we need to overcome safety issues when applying MVs and exosomes as modes for drug delivery in cancer. The use of EVs in medicine is still in its infancy, and there are many potholes to cover, but their utility as diagnostic tools or as delivery vehicles in glioblastoma is an unmet challenge.

## Author Contributions

AS conceptualized the review and wrote it. AM and DK prepared the draft and figures; contributed equally to this review. SP helped in preparation of the draft.

## Conflict of Interest Statement

The authors declare that the research was conducted in the absence of any commercial or financial relationships that could be construed as a potential conflict of interest.
